# The Impacts of *Lactobacillus delbrueckii* and *Lactobacillus rhamnosus* to Promote In Vitro Anti‐Inflammatory Profile of RA‐Macrophages

**DOI:** 10.1002/fsn3.70068

**Published:** 2025-03-16

**Authors:** Parisa Ahmadi, Mahmoud Mahmoudi, Houshang Rafatpanah, Zahra Rezaieyazdi, Maryam Ahmadi‐Khorram, Zahra Javanmardi, Nafiseh Sadat Tabasi, Seyed‐Alireza Esmaeili

**Affiliations:** ^1^ Immunology Research Center Mashhad University of Medical Sciences Mashhad Iran; ^2^ Immunology Department, Faculty of Medicine Mashhad University of Medical Sciences Mashhad Iran; ^3^ Student Research Committee Mashhad University of Medical Sciences Mashhad Iran; ^4^ Division of Inflammation and Inflammatory Diseases, Immunology Research Centre Mashhad University of Medical Sciences Mashhad Iran; ^5^ Rheumatic Diseases Research Center Mashhad University of Medical Sciences Mashhad Iran; ^6^ Department of Nutrition, Faculty of Medicine Mashhad University of Medical Sciences Mashhad Iran

**Keywords:** cytokines, *Lactobacillus delbrueckii*, *Lactobacillus rhamnosus*, M0 macrophage, M1 macrophage, M2 macrophage, probiotic, rheumatoid arthritis

## Abstract

Rheumatoid arthritis (RA) is a prevalent and debilitating autoimmune disease. Numerous studies have demonstrated promising results regarding the use of probiotics as a therapeutic approach to alleviate RA symptoms. This study isolated monocytes from the PBMCs of RA patients and healthy donors. These monocytes were then differentiated into macrophages and divided into five groups: untreated, LPS‐treated, *L. delbrueckii* (Del)‐treated, *L. rhamnosus* (Ram)‐treated, and a mixed treatment group. Three macrophage subpopulations—M0, M1, and M2—were identified in all treatment groups, with variations observed in the population percentages of each subpopulation and the expression levels of CD14, CD80, and HLA‐DR. Flow cytometry results indicated that, compared to the untreated and LPS‐treated groups, treatment with probiotic bacteria (Del, Ram, and Mix) stimulated the polarization of macrophages toward the M2 phenotype while suppressing the percentage of the M1 population. Additionally, the expression of CD14, a Pathogen‐Associated Molecular Pattern (PAMP) and phagocytosis‐inducing receptor, was significantly reduced in the probiotic‐treated groups. Probiotic treatment also profoundly influenced antigen presentation by suppressing CD80, a ligand for the CD28 co‐stimulatory marker on T cells, and HLA‐DR, which presents antigens to the T cell receptors of Th4 cells. Interestingly, quantitative real‐time PCR results indicated that probiotic treatment of macrophages significantly increased the expression of IL‐10 and TGF‐β, both anti‐inflammatory cytokines, while significantly decreasing the expression of inflammatory cytokines, including IL‐12, IL‐1β, and TNF‐α, in both healthy controls and RA patients. It seems that these probiotics may have a regulatory effect on macrophages, affecting their polarization, antigen presentation patterns, phagocytosis, and cytokine secretion profiles. This suggests that these probiotics may have therapeutic and prophylactic effects on RA.

## Graphical abstract

PBMCs were first separated from blood using the Ficoll method. Then, monocytes were isolated from PBMC using the plastic adherence method to differentiate them into macrophages within 5 days. Subsequently, they were treated with LPS, *Lactobacillus delbrueckii* (Del), *Lactobacillus rhamnosus* (Ram), and a mix of them. The probiotic effects on macrophages were assessed using qRT‐PCR and flow cytometry. Treating macrophages with *L. delbrueckii* and *L. rhamnosus* significantly alters their capability to stimulate CD4+ T cells. Probiotic bacteria not only reduce the expression of HLA‐DR, which is essential for T cell receptor (TCR) signaling (the first stimulatory signal for T cells) but also decrease the CD80 co‐stimulatory marker on macrophages, which interacts with CD28 on T cells (the second stimulatory signal for T cells). Additionally, these probiotic bacteria lower the expression of CD14 on macrophages, a receptor involved in receptor‐mediated phagocytosis. Significantly, treatment with these probiotics enhances the production of anti‐inflammatory cytokines, including IL‐10 and TGF‐β, while simultaneously diminishing the expression of pro‐inflammatory cytokines such as IL‐12, IL‐1β, and TNF‐α in macrophages. These findings suggest a polarization of macrophages toward the M2 phenotype in both RA patients and healthy individuals. LPS‐treated groups, treatment with probiotic bacteria (Del, Ram, and Mix).

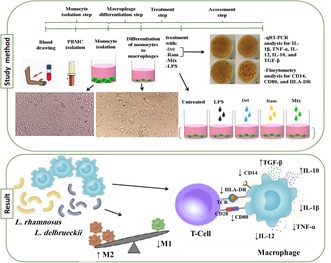

## 1 Introduction

Rheumatoid arthritis (RA) is an autoimmune inflammatory disease that affects approximately 1% of the global population (Ahmadi et al. 2023). It is associated with chronic, disabling pain and a variety of intra‐ and extra‐articular manifestations (Cojocaru et al. 2010). Current treatment for rheumatoid arthritis relies on immunosuppressive medications and analgesics, which can result in drug resistance, intolerance, and other adverse effects (Lai and Lan 1998). Therefore, an approach that emphasizes immunoregulation rather than immunosuppression may be more beneficial for the prevention and management of RA. Numerous studies have demonstrated that tolerogenic probiotics, such as *Lactobacillus* spp., can regulate the immune system in autoimmune diseases like RA through mechanisms such as competition and elimination of pathogenic bacteria, the production of nutrients like vitamins, and their anti‐inflammatory properties (Sayaf et al. 2025; Taherkhani et al. 2024; Javanmardi et al. 2024; Liu et al. 2018; Oliviero and Spinella 2020). A study by Sun et al. (2019) found that RA patients had a significantly limited population of *Lactobacillus* compared to healthy individuals. Other research has shown promising results in managing RA through the administration of specific *Lactobacillus* strains, such as *Lactobacillus casei* and *Lactobacillus rhamnosus* (Fernández‐Llanio Comella et al. 2016; Hatakka et al. 2003).

In the early stages of RA, innate immunity plays a crucial role, with macrophages serving as the most important innate immune cells. Macrophages can secrete pro‐ or anti‐inflammatory cytokines, present various antigens and autoantigens to T cells, and phagocytose immune complexes and microbes (Allahyari et al. 2020; Tardito et al. 2019; Shapouri‐Moghaddam et al. 2018). Macrophage polarization significantly influences the progression of RA. M1 macrophages secrete cytokines such as IL‐1, IL‐6, IL‐23, and TNF‐α, promote the differentiation of T cells into Th1 and Th17, stimulate osteoclasts and synovial cells, and attract monocytes and neutrophils to specific sites (Tardito et al. 2019; Hoseinzadeh et al. 2023; Hosseini et al. 2024). Conversely, M2 macrophages promote the differentiation of T cells into regulatory T cells (T regs) through their anti‐inflammatory cytokines (Tardito et al. 2019; Shapouri‐Moghaddam et al. 2018). Studies have demonstrated that *Lactobacillus* probiotics can affect macrophage polarization. For instance, research conducted by Sichetti et al. (2018) revealed that treating THP‐1 macrophages with a probiotic serum containing *L. rhamnosus* promoted M2 macrophage polarization. A study by Kim et al. (2020) similarly demonstrated that extracellular vesicles derived from *Lactobacillus plantarum* facilitated the differentiation of THP‐1 macrophages into anti‐inflammatory M2 macrophages. Additional research has shown that pretreatment of macrophages with *L. rhamnosus* , *L. acidophilus* , and *L. casei* reduces M1 markers, including Dectin‐1, Toll‐Like Receptor‐2 (TLR‐2), and TLR‐4, while increasing the secretion of IL‐10 (Matsubara et al. 2017).

Given the critical role of macrophages in phagocytosis, studies have investigated the effects of probiotics on these immune cells by monitoring the expression of CD14. This marker is a pattern recognition receptor (PAMP receptor) inducing phagocytosis and influencing cell granularity, which serves as an indicator of cellular organelles, including phagosomes (Wu et al. 2019). Meanwhile, the effects of probiotics on macrophage functions, such as antigen presentation and T cell stimulation via CD80—a ligand for the CD28 co‐stimulatory marker on T cells—and the MHC class II molecule HLA‐DR were also analyzed. Additionally, the influence of probiotics on phagocytosis and the production of anti‐inflammatory cytokines (IL‐10, TGF‐β) and pro‐inflammatory cytokines (IL‐1β, IL‐12, TNF‐α) was evaluated. This study focuses on the effects of the tolerogenic probiotics *Lactobacillus delbrueckii* and *L. rhamnosus* on macrophage polarization and their impact on key macrophage functions, including phagocytosis and the cytokine secretion profile in RA patients and healthy controls (HC).

## 2 Materials

### 2.1 Probiotics and Their Mediums

Lyophilized powder of *L. rhamnosus* (ATCC: 9595) and *L. delbrueckii* subsp. *lactis* (PTCC: 1743DSM) was purchased from the Pasteur Institute of Iran (IPI) and the Iranian Research Organization for Science and Technology (IROST), respectively. Additionally, Man, Rogosa, Sharpe (MRS) broth (QUILAB, Cat. No. 65‐5198), MRS agar (QUILAB, Cat. No. QB‐65‐9306), and glycerol were provided (Figure S1).

### 2.2 Monocyte Isolation, Enumeration, and Culture Reagents

Ficoll‐Paque (SIGMA, USA, Cat. No. F2637); Dulbecco's Modified Eagle's Medium (DMEM) with 1 g/L D‐glucose and pyruvate (GIBCO, USA, Cat. No. 31600‐083), heat‐inactivated at 56°C for 30 min; fetal bovine serum (Biowest, South America Origin, USA, Cat. No. S181A‐100); 0.25% trypsin/1 mM EDTA (GIBCO, USA, Cat. No. 27250‐018); L‐glutamine (SIGMA, Cat. No. G3126‐100g); streptomycin salt (SIGMA, Cat. No. S1277‐5G); penicillin G potassium salt (SIGMA, Cat. No. P7794‐1MU); lipopolysaccharides from *Escherichia coli* 0111:B4 (SIGMA, Cat. No. L4391‐1MG); NaHCO3 (SIGMA, Cat. No. S5761); phosphate‐buffered saline (PBS, pH 7.4); Trypan blue (SIGMA); and complete RPMI supplemented with 100 IU/mL penicillin, 100 μg/mL streptomycin, and 2 mM L‐glutamine. To prepare 100 mL complete DMEM medium (with 10% FBS), 2 mM L‐glutamine, 100 mg/μL penicillin, and 100 mg/μL streptomycin, 3.7 g/L NaHCO3, and 10% FBS were combined, and then the volume was adjusted to 100 mL by adding DMEM.

### 2.3 Flow Cytometry Reagents

CD14‐R‐PE (IQProducts, the Netherlands, Cat. No. IQP‐143R), anti‐HLA‐DR‐R‐PE (IQProducts, Cat. No. IQP‐134R), and CD80 FITC (Bio‐Rad, UK, Cat. No. MCA2071F).

### 2.4 PCR Reagents

cDNA Synthesis Kit (Pastors, Cat. No. A101162), SYBR Green rT‐PCR Kit (Amplicon, Cat. No. A325402), ethanol, isopropanol, chloroform, and TRI Reagent.

## 3 Methods

### 3.1 Subjects and Ethics Statement

Blood samples were collected from healthy donors and patients recently diagnosed with rheumatoid arthritis who had not received immunosuppressive or anti‐inflammatory treatment. All volunteers who participated in the study provided written informed consent, and the study was approved by the ethics committee of Mashhad University of Medical Sciences (Ethical code: IR.MUMS.MEDICAL.REC.1399.814).

### 3.2 Bacterial Strain Preparation

In the first step of bacterial preparation, both lyophilized bacterial powders were cultured separately in MRS broth for 1 h at 37°C under microaerobic conditions. In the second step, the probiotics were cultured on two bacteriological plastic (BP) Petri dishes containing MRS agar for 24 h at 37°C under microaerobic conditions. The strains were then preserved as glycerol (20% v/v) stocks at −80°C until they were recultured in MRS medium for treatment with cell cultures.

### 3.3 Isolation of PBMCs From Human Peripheral Blood

Blood samples (10 mL) were collected from rheumatoid arthritis patients and healthy volunteers in separate lithium/heparinized tubes. PBMCs were isolated from the buffy coat layer using Ficoll‐Paque density gradient centrifugation. The mononuclear cell fraction at the interface layer was carefully transferred to a sterile tube and washed three times with phosphate‐buffered saline (PBS, pH 7.4) to eliminate platelets.

### 3.4 PBMCs Counting and Adjusting in Cell Culture Plates

The washed PBMC plate was resuspended in 5 mL of complete DMEM medium during the primary dilution step. The Trypan Blue Exclusion Assay determined the number of cells in the primary diluted suspension. A volume of 20 μL of the PBMC suspension was mixed with an equal volume of 0.4% Trypan Blue solution, and the unstained cells were counted using a hemocytometer and microscopic examination. Based on the cell count, ranging from 0.4 to 0.8 × 10^6^ cells/mL, an additional complete medium was added to achieve the desired seeding cell density of 0.2 × 10^6^ cells per well in the secondary dilution step. Finally, the diluted PBMCs were added to 10 wells of a 24‐well polystyrene tissue culture plate.

### 3.5 Isolation of Monocytes From PBMCs and Treatment

The plastic adherence method was used to isolate monocytes from PBMCs. Cultures were incubated at 37°C in a cell culture incubator under humidified conditions with 5% CO_2_ for 2 h to promote monocyte adherence. Following this incubation period, the monocytes adhered to the inner surface of the wells. Non‐adherent peripheral blood mononuclear cells, including T, B, and natural killer (NK) cells, were removed by aspirating the DMEM. To further purify the adherent monocytes, they were gently washed three times with pre‐warmed phosphate‐buffered saline (PBS). The purity of the isolated monocytes was subsequently confirmed by flow cytometry using CD14‐R‐PE antibodies.

### 3.6 Monocyte Treatment and Culture

Subsequently, 2 mL of complete DMEM was added. The cultures were divided into untreated and four different treatment groups (consisting of LPS, *L. delbrueckii* , *L. rhamnosus* , and a mixture of both probiotics). For the probiotic treatment, the glycerol stocks of the probiotics were centrifuged, the supernatant was discarded, and the cell plate was washed three times with PBS to remove any residual glycerol. Before the treatment of the wells, the probiotic bacteria were pre‐cultured in MRS broth and MRS agar for 2 and 24 h, respectively. Fresh cultures of probiotics were then washed and diluted for monocyte treatment. LPS (100 ng/mL), *L. delbrueckii* (2 × 10^6^ bacteria/well), *L. rhamnosus* (2 × 10^6^ bacteria/well), and the mixed treatment (1 × 10^6^ *L. delbrueckii* + 1 × 106 *L. rhamnosus* bacteria/well) were added to their respective wells. The treated monocytes were incubated for 5 days to generate mature macrophages. The culture medium was discarded every 3 days, and the adherent cells were gently washed with warmed PBS. After 5 days of cell culture, the adherent monocytes were differentiated into macrophages according to their specific treatments. They were then harvested from the wells by adding 1 mL of 0.25% trypsin–EDTA to each well and incubating for 2 min at room temperature. The trypsin was neutralized by adding 1.5 mL of complete medium, and the cells were collected for further study.

### 3.7 Flow Cytometry

The expression of surface markers CD14, CD80, and HLA‐DR on harvested cells was analyzed using flow cytometry with fluorochrome‐conjugated monoclonal antibodies: CD14‐R‐PE, anti‐HLA‐DR‐R‐PE, and CD80‐FITC. This analysis aimed to confirm the differentiation and expression of these surface markers. For the flow cytometry procedure, harvested cells were washed with a cell wash buffer, stained with the fluorochrome‐conjugated monoclonal antibodies, and incubated on ice for 30 min. The cells were washed twice to remove any unbound antibodies and were resuspended in the cell wash buffer.

Flow cytometry data were acquired using a FACSCalibur flow cytometer (Becton Dickinson) and analyzed with CellQuest Pro (BD Biosciences, San Jose, CA) or Flowjo 7.6.2 software (Tree Star, Ashland, OR).

### 3.8 RNA Extraction and Complementary DNA Synthesis

Total RNA was extracted from macrophage cells using TRIzol reagent, following the manufacturer's instructions, and examined with a NanoDrop One spectrophotometer. Complementary DNA (cDNA) was synthesized from 1 μg of RNA according to the instructions provided in the cDNA synthesis kit. The synthesized cDNA was then quantified using a NanoDrop One spectrophotometer.

### 3.9 Quantitative Real‐Time Polymerase Chain Reaction (qRT‐PCR)–SYBR Green Method

**FIGURE 1:**
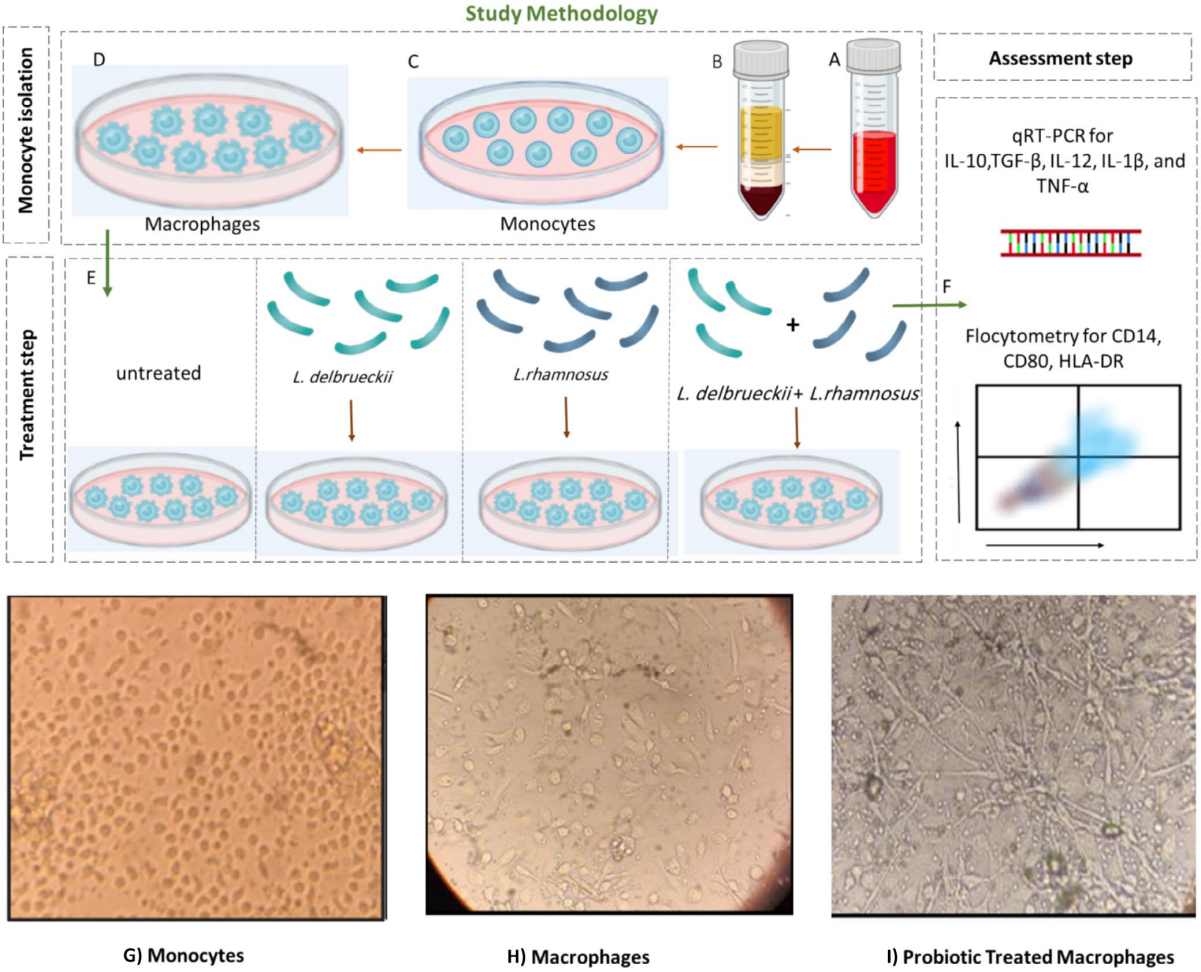
Steps and general study methodology. In the monocyte isolation step: (A) Blood was collected from three infected patients and three healthy individuals. (B) Their PBMCs were isolated using the Ficoll method. (C) Monocytes were adhered to the surface of the wells using an adhesion method. (D) Within 5 days, the monocytes differentiated into macrophages. (E) After these 5 days, the macrophages were divided into five groups: Untreated, treated with LPS, *Lactobacillus delbrueckii* , *Lactobacillus rhamnosus* , and a mixture of both bacteria for 48 h. In the evaluation step: (F) After treatment, the treated macrophage cells were assessed for the expression of CD80, HLA‐DR, and CD14 markers by flow cytometry, as well as the expression of IL‐1, IL‐10, IL‐12, TNF‐α, and TGF‐β cytokines using the real‐time PCR method. (G) Morphology of monocytes isolated from PBMCs. (H) Morphology of macrophages differentiated from monocytes after 5 days. (I) Morphology of macrophages 2 days after treatment with a mixture of probiotics ( *L. delbrueckii* and *L. rhamnosus* ).

Quantitative real‐time polymerase chain reaction (qRT‐PCR) was conducted in 15 mL reactions containing 1 μL of cDNA, sterile deionized water, a 10 μL mixture of both primers, and 5 μL of SYBR Green Master Mix. The primers are detailed in Table 1. The reaction used a LightCycler 96 (Roche, Basel, Switzerland). To normalize the cycle threshold (*C*_t_) values of the genes of interest, glyceraldehyde‐3‐phosphate dehydrogenase (GAPDH) was employed as a housekeeping gene. Following normalization, relative expression levels were calculated using the fold‐change ($2^{-\Delta C_{t}}$) method (Figure 1).

**TABLE 1:** Forward and reverse primers were applied in this study.

Genes	Forward	Reverse	Accession number
*GAPDH*	GTCTCCTCTGACTTCAACAGCG (*n* = 22)	ACCACCCTGTTGCTGTAGCCAA (*n* = 22)	NM_001256799
*IL‐10*	TCTCCGAGATGCCTTCAGCAGA (*n* = 22)	TCAGACAAGGCTTGGCAACCCA (*n* = 22)	NM_000572
*IL‐12*	GACATTCTGCGTTCAGGTCCAG (*n* = 22)	CATTTTTGCGGCAGATGACCGTG (*n* = 22)	NM_002187
*TGF‐β*	TACCTGAACCCGTGTTGCTCTC (*n* = 22)	GTTGCTGAGGTATCGCCAGGAA (*n* = 22)	NM_000660
*TNF‐α*	CTCTTCTGCCTGCTGCACTTTG (*n* = 22)	ATGGGCTACAGGCTTGTCACTC (*n* = 22)	NM_000594
*IL‐1β*	CCACAGACCTTCCAGGAGAATG (*n* = 22)	GTGCAGTTCAGTGATCGTACAGG (*n* = 22)	NM_000576

Abbreviations: GAPDH, glyceraldehyde 3‐phosphate dehydrogenase; IFN‐γ, interferon‐gamma; IL‐12, interleukin 12; TGF‐β, transforming growth factor beta.

### 3.10 Statistical Analyses

All data frequencies were compared using one‐way repeated measures ANOVA with Tukey's post hoc test and a two‐way ANOVA. Statistical analyses were conducted using GraphPad Prism software (version 6). *p* ≤ 0.05 were considered statistically significant.

## 4 Results

### 4.1 Subjects and Ethics Statement

Three healthy donors and three recently diagnosed RA patients participated in this study. All RA patients tested positive for rheumatoid factors (RF) and anti‐cyclic citrullinated peptide (anti‐CCP) antibodies. In contrast, healthy donors were tested negative for both RF (Figure S2) and anti‐CCP antibodies. Monocytes were isolated from the PBMCs of both healthy and RA donors, and the purity of the isolated monocytes was confirmed using flow cytometry.

### 4.2 Confirmation of PBMC Viability

**FIGURE 2:**
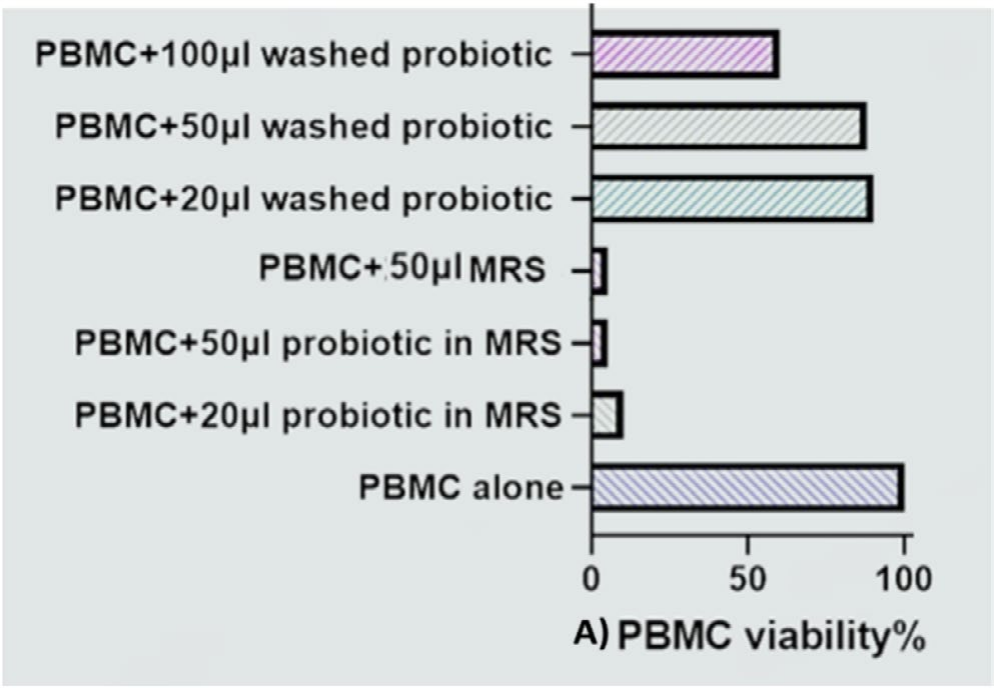
The viability percentage of PBMCs in various suspensions shows that MRS is a toxic suspension for PBMCs, resulting in cell death. However, probiotics alone are not cytotoxic to PBMCs that contain monocytes.

Trypan blue staining of PBMCs indicated that MRS broth, the culture medium for *Lactobacillus* species, was highly toxic to PBMCs. The addition of 20 and 50 μL of probiotic suspension in MRS broth, as well as 50 μL of MRS broth without probiotics, resulted in a significant decrease in PBMC viability, with percentages of 10%, 4%, and 5%, respectively (Figure 2). Conversely, washing the probiotics suspended in MRS broth did not affect their viability, demonstrating that the probiotic bacteria exhibited no cytotoxicity toward PBMCs. The viability percentages for 20, 50, and 100 μL of washed probiotic suspension were 90%, 88%, and 60%, respectively (Figure 2).

### 4.3 Assessment of Monocyte Isolation Method

**FIGURE 3:**
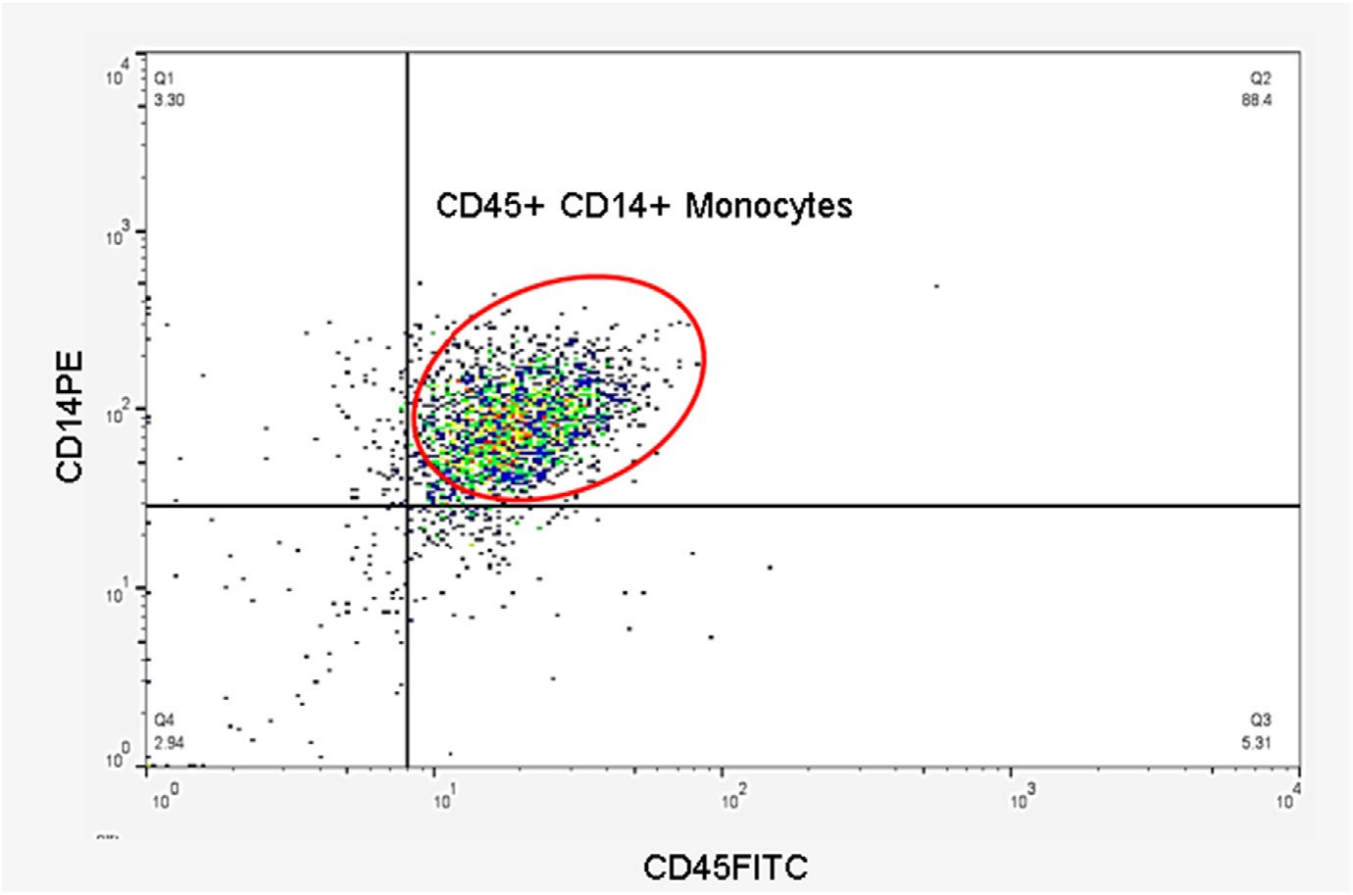
Monocytes (CD45+ and CD14+ cells) were isolated from the peripheral blood mononuclear cells by the plastic adherence method with a purity of 88.4%. In this method, after separating the buffy coat layer by the Ficoll‐Paque method, these cells were cultured in DMEM cell culture medium and incubated for 2 h in a humid incubator. In this way, monocytes adhered to cell culture wells, but other cells in PBMC, including lymphocytes and neutrophils, were suspended in the DMEM medium. Then, by aspiration and washing of monocytes with PBS, monocytes were isolated.

Flow cytometric analysis of the separated buffy coat layer containing PBMCs showed that CD14+ CD45+ cells, which include monocytes, constituted 21.5% of the PBMC population. The evaluation of the efficacy of the plastic adherence method in our study demonstrated that after 2 h of incubation and subsequent washing of the PBMCs, 88.4% of the isolated cells were identified as CD14+ CD45+ monocytes (Figure 3).

### 4.4 Flow Cytometry Results

After 5 days in culture, isolated human monocytes spontaneously differentiated into macrophages. To investigate the tolerogenic effects of two different probiotic bacteria, *L. lactis* and *L. delbrueckii* , on the cell markers and cytokines of monocyte‐derived macrophages, the macrophages were cultured for 48 h and subsequently treated with *L. lactis* , *L. delbrueckii* , or a combination of both. These treated macrophages were then evaluated by flow cytometry and PCR methods. The results were compared with inflammatory conditions induced by LPS coculture of monocyte‐derived macrophages (MDM) for 48 h and with the untreated status.

### 4.5 Flow Cytometry Analysis and Gating Strategy of Macrophages

**FIGURE 4:**
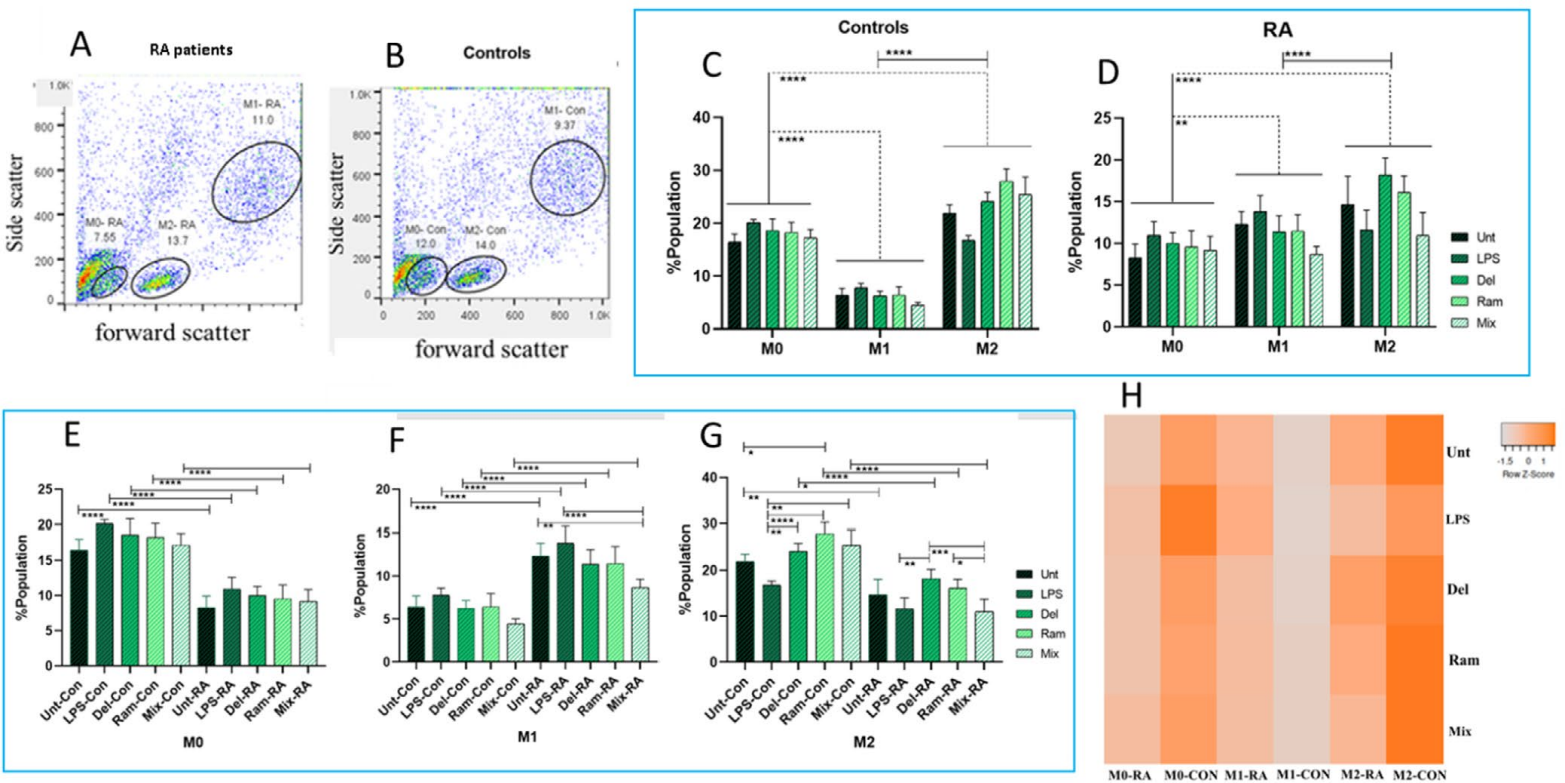
The comparison of population percentages of distinct macrophage subtypes (M0, M1, and M2) in healthy controls (HC) and individuals with RA using flow cytometry. (A, B) Gating of M0, M1, and M2 subpopulations of macrophages in HC and RA. (C) The comparison of the population percentages of macrophage subpopulations in HC and RA; in HC, M2 macrophages had the largest population, while M1 macrophages represented the smallest. (D) In RA patients, M2 macrophages had the largest percentage of the population, followed by M1 macrophages, which were significantly more than M0 macrophages. (E) Comparison between counterpart treatment groups of RA and HC; The M0 macrophages of HC had higher levels of M0 macrophages than RA patients in all the treatment groups, including untreated, LPS, Del Ram, and mix. (F) In the M1 subpopulation, comparisons among the treatment groups indicated that HC had significantly lower M1 macrophages than RA patients. Furthermore, comparisons among the treatment groups of RA patients showed that the Mix group had significantly lower levels of M1 macrophages than both the untreated and LPS‐treated groups. (G) Among the M2 subpopulations, HCs had more M2 macrophages than RA patients. Furthermore, in HCs, the treated group had significantly fewer M2 macrophages than all of the probiotic‐treated groups. In RA patients, the LPS‐treated group exhibited a significantly lower M2 population than the Del‐treated group. Meanwhile, in RA patients, the Mix group had a significantly lower M2 population than both the Del and Ram groups. (H) A heat map visualizing the percentage of M0, M1, and M2 macrophages in different treatment groups of RA patients and HCs. Each value is presented as the mean ± standard deviation (SD). Analysis of variance tests, including two‐way ANOVA, Tukey's post hoc test, and one‐way ANOVA. *p < 0.05, **p ≤ 0.01, ***p ≤ 0.001, and ****p < 0.0001.

After 7 days of culture (5 days for macrophage differentiation plus 2 days of probiotic treatment), a flow cytometric assessment of macrophages was conducted. Forward scatter (FSC) and side scatter (SSC) characteristics indicated cell size and granularity, respectively. Small debris with low SSC and FSC, located in the lower‐left corner of the dot plot, was excluded from the analysis. The remaining cells represented the general macrophage population, which exhibited a larger size and granularity than the debris. Doublets (clumps of cells with increased area but similar height compared to single cells) were excluded from the gating strategy to ensure that only individual macrophage cells were included in the study. Within the general macrophage population, three distinct subpopulations were gated based on surface marker levels and cell behavior: (A) M0 macrophages, characterized by low SSC (50–200), low FSC (150–300), and intermediate overall expression of HLA‐DR, CD80, and CD14; (B) M2 polarized macrophages, identified by intermediate FSC (300–550); and (C) M1 polarized macrophages, defined by the highest SSC (300–650), FSC (650–1000), and general expression of HLA‐DR, CD80, and CD14 (Figure 4).

### 4.6 Quantitative Analysis of the Macrophage Population Among the Treatment Groups

At a glance, the general tendency of monocytes to differentiate into the three classes of macrophages differs between RA patients and HC. In both groups, M2 macrophages constituted the largest population. Compared to the M1 and M0 macrophages, M2 cells exhibited significantly higher differentiation (*p*_M0 vs. M2_ < 0.0001, *p*_M1 vs. M2_ < 0.0001). Further comparisons between M0 and M1 macrophages revealed that the M0 population was the second largest macrophage population in HC, while M1 macrophages were the smallest (*p*_M0 vs. M1_ < 0.0001). Conversely, among the RA patients, M1 macrophages represented the second largest population, and M0 macrophages were the smallest (*p*_M0 vs. M1_ = 0.0010) (Figure 4).

Further comparisons between RA patients and HC showed that in all treatment groups, the M1 population was significantly higher in RA patients than in HC (*p*_Unt‐RA vs. Unt‐Con_ < 0.0001, *p*_LPS‐RA vs. LPS‐Con_ < 0.0001, *p*_Del‐RA vs. Del‐Con_ < 0.0001, *p*_Ram‐RA vs. Ram‐Con_ < 0.0001, *p*_Mix‐RA vs. Mix‐Con_ < 0.0001) (Figure 4). Conversely, in all treatment groups, the controls exhibited significantly higher levels of differentiation to M0 macrophages compared to RA patients (*p*_Unt‐RA vs. Unt‐Con_ < 0.0001, *p*_LPS‐RA vs. LPS‐Con_ < 0.0001, *p*_Del‐RA vs. Del‐Con_ < 0.0001, *p*_Ram‐RA vs. Ram‐Con_ < 0.0001, *p*_Mix‐RA vs. Mix‐Con_ < 0.0001) (Figure 4). Similar to the M0 population, controls also had a significantly higher population of M2 macrophages than RA patients across all treatment groups (*p*_Unt‐RA vs. Unt‐Con_ = 0.0015, *p*_LPS‐RA vs. LPS‐Con_ = 0.001, *p*_Del‐RA vs. Del‐Con_ = 0.0164, *p*_Ram‐RA vs. Ram‐Con_ < 0.0001, *p*_Mix‐RA vs. Mix‐Con_ < 0.0001) (Figure 4).

### 4.7 Analysis of Cell Surface Protein Markers

#### 4.7.1 The Expression Levels of CD14, CD80, and HLA‐DR in M0, M1, and M2 Macrophage Subtypes

**FIGURE 5:**
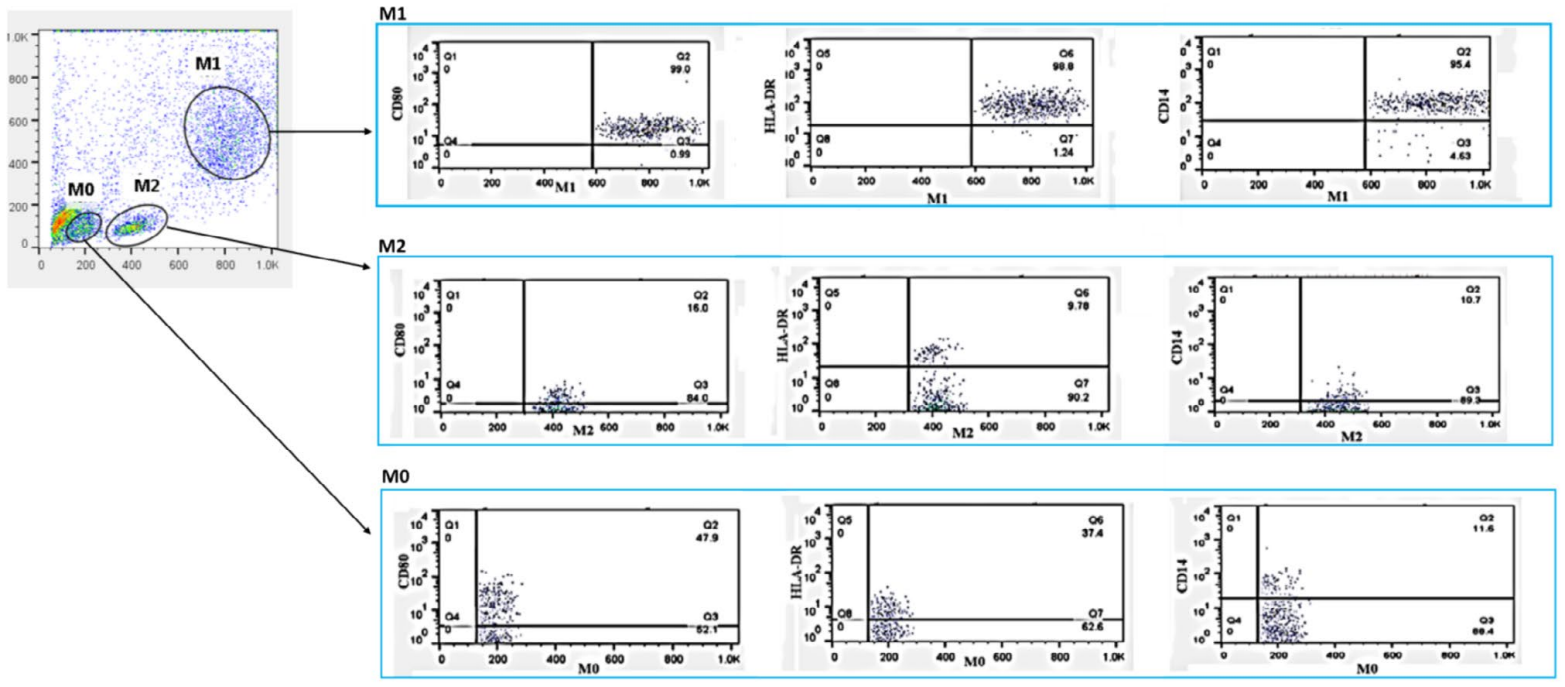
The expression levels of CD14, CD80, and HLA‐DR in M0, M1, and M2 macrophage subpopulations were analyzed using flow cytometry. After gating the three macrophage subpopulations based on side and forward scatter, these subpopulations were categorized into M0, M1, and M2 according to the expression levels of CD14, CD80, and HLA‐DR. The population exhibiting the highest levels of CD80, CD14, and HLA‐DR was designated as the M1 subpopulation. The population with the lowest levels of these markers was labeled as the M2 subpopulation, while the macrophage subpopulation expressing intermediate levels of these markers was considered the M0 subpopulation.

**FIGURE 6:**
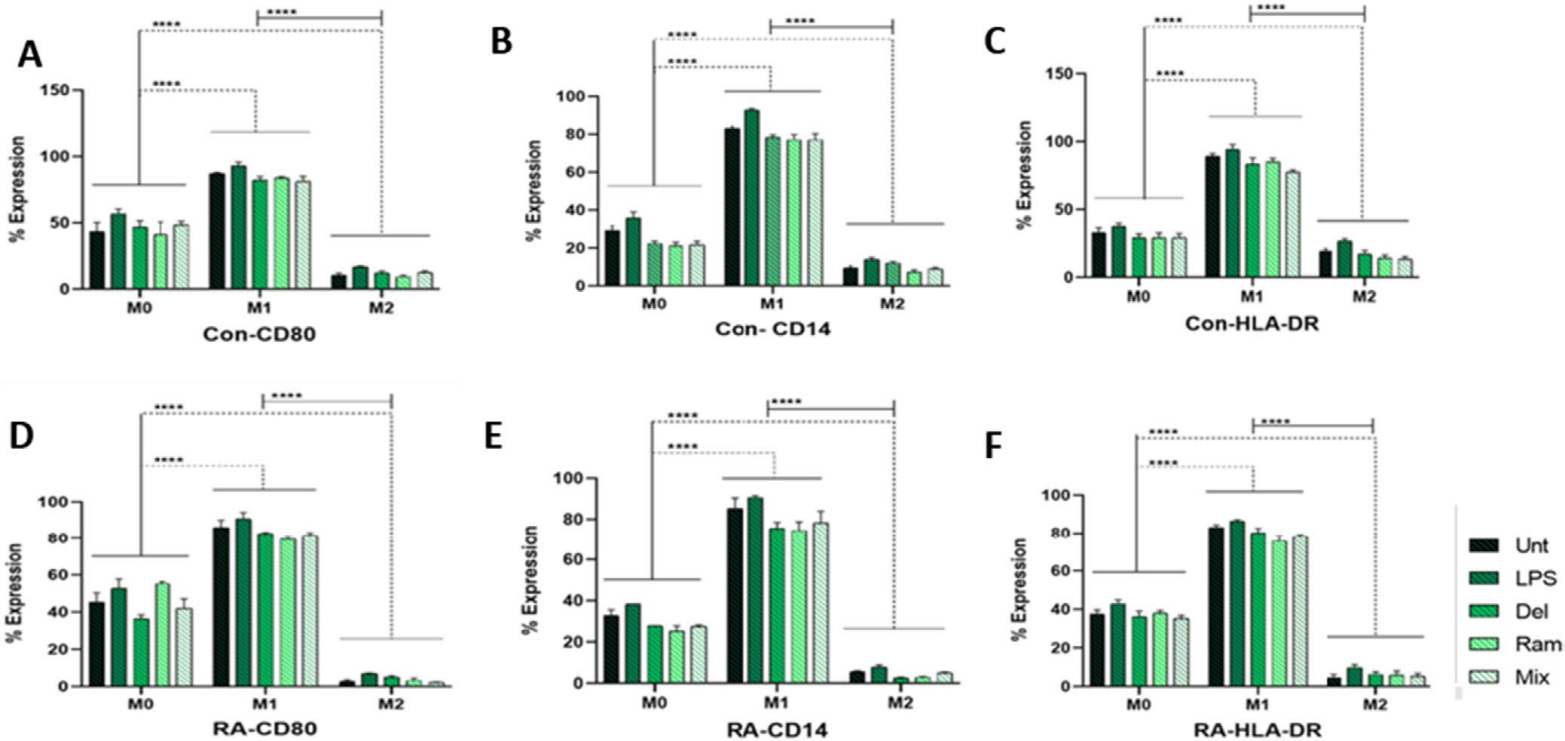
Comparison of CD14, CD80, and HLA‐DR Expression Levels Among Distinct Macrophage Subtypes (M0, M1, and M2) in Healthy Controls (HC) and Patients with Rheumatoid Arthritis (RA). Overall, the comparison of expression levels of CD80 (A, D), CD14 (B, E), and HLA‐DR (C, F) between healthy donors and RA patients revealed that the M1 subpopulation expressed significantly higher levels of these markers compared to M0 and M2 macrophages. In contrast, the M2 subpopulation exhibited the lowest levels of these markers, which were significantly lower than those of M0 and M1 macrophages. Untreated (Unt), *Lactobacillus delbrueckii* (Del), and *Lactobacillus rhamnosus* (Ram). Each value is presented as the mean ± standard deviation (SD). Different letters in the figure indicate significant differences between groups (*p* < 0.05). ^#^One‐way analysis of variance (ANOVA) test; ****p < 0.0001.

Flow cytometry analysis of cell surface markers, including CD14, CD80, and HLA‐DR, in both RA patients and healthy controls, revealed significant differences in expression levels among the three macrophage populations. In both HC and RA patients, the M1 population exhibited significantly higher levels of these cell markers compared to M2 (*p* < 0.0001) and M0 macrophages (*p* < 0.0001). Further comparisons between the M0 and M2 subpopulations of macrophages indicated that M2 macrophages expressed significantly lower levels of CD14, CD80, and HLA‐DR than M0 macrophages (*p* < 0.0001) (Figures 5 and 6).

#### 4.7.2 The Expression Levels of CD14, CD80, and HLA‐DR Surface Markers in M0 Macrophage Population of Healthy and RA Groups

**FIGURE 7:**
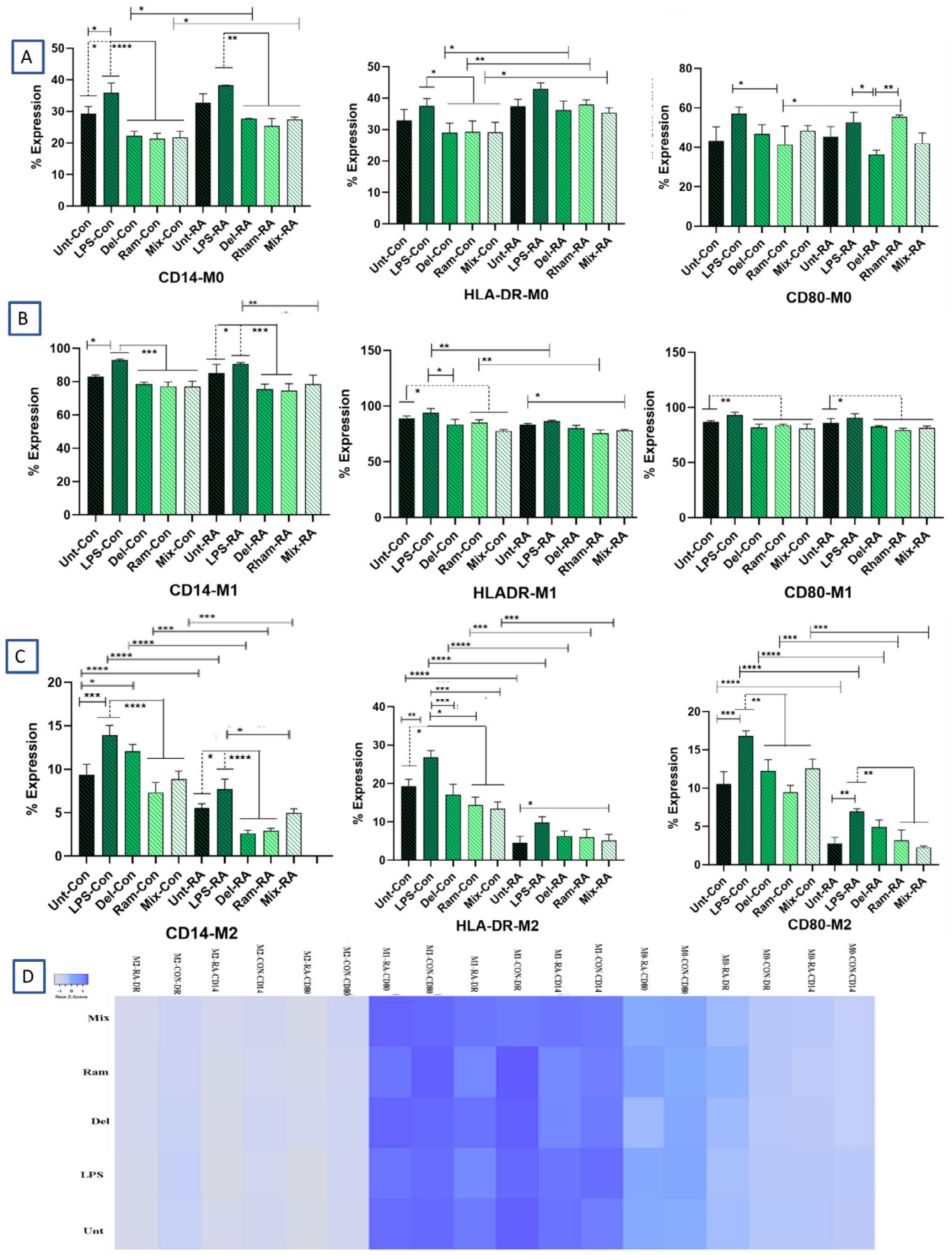
The flow cytometry results showing the expression of CD14, CD80, and HLA‐DR on rheumatoid arthritis (RA) and healthy control (HCs) macrophages treated with lipopolysaccharide (LPS) and probiotics (Ram, Del, and Mix). (A) (M0 Macrophages) Except CD80 expression in the Del and Mix groups compared to the untreated controls, as well as Ram versus untreated and LPS, probiotic treatment resulted in decreased expression of CD14, CD80, and HLA‐DR (significant reductions are indicated in the figure). A comparison between the corresponding treatment groups of RA and control macrophages revealed that RA patients exhibited significantly higher expression levels of CD14, CD80, and HLA‐DR in certain instances, including Del and Mix for CD14 expression, all probiotic groups for HLA‐DR, and Ram treatment for CD80. (B) (M1 Macrophages) Probiotic treatments decreased the expression of CD14, CD80, and HLA‐DR in both RA and healthy control macrophages, with significant reductions indicated in the figure. The comparison between counterpart treatment groups of RA and control macrophages revealed that RA patients exhibited significantly higher expression of Ram and LPS‐treated groups for HLA‐DR. The remaining comparisons showed similar expression levels of these markers between RA and control groups. (C) (M2 Macrophages) Except for the untreated versus Del comparison in controls for CD14, the untreated versus probiotics comparisons in RA for HLA‐DR, as well as the Del and Mix versus untreated comparison for CD80, and the Del and Ram versus untreated comparison in RA for CD80, probiotic treatment resulted in decreased expression of CD14, CD80, and HLA‐DR (significant reductions are indicated in the figure). The comparison between the corresponding treatment groups of RA and control macrophages revealed that controls expressed significantly higher levels of CD14, CD80, and HLA‐DR than RA macrophages. (D) A heat map visualizes CD14, CD80, and HLA‐DR expression levels on macrophages across various treatment groups of RA patients and HCs. The bars are presented with mean ± standard deviation (SD) values. Different letters in the figure indicate significant differences between groups (*p* < 0.05). The statistical analysis was conducted using a one‐way analysis of variance (ANOVA) test; *p < 0.05, **p ≤ 0.01, ***p ≤ 0.001, and ****p < 0.0001.

Statistical analysis showed a significant decrease in the expression of the CD14 marker in all probiotic groups (Del, Ram, and Mix) compared to the LPS group in both healthy and RA patient‐derived macrophages (in controls, *p*_Del vs. LPS_ < 0.0001, *p*_Ram vs. LPS_ < 0.0001, *p*_Mix vs. LPS_ < 0.0001 and in RA patients, *p*_Del vs. LPS_ < 0.0034, *p*_Ram vs. LPS_ = 0.0005, *p*_Mix vs. LPS_ = 0.0027) (Figure 7A). LPS treatment increased CD 14 expression in both HC and RA, but it was significant only in controls (*p*_LPS vs. Unt_ = 0.0290) (Figure 7A). Comparison between untreated and probiotic‐treated groups showed that while probiotic treatment decreased CD14 expression in both RA patients and HC, it was statistically significant only in controls (*p*_LPS vs. Unt_ = 0.0290, *p*_Del vs. Unt_ = 0.0233, *p*_Ram vs. Unt_ = 0.0078, *p*_Mix vs. Unt_ = 0.0121) (Figure 7A). Regarding HLA‐DR expression, in the control group, probiotic‐treated macrophages exhibited significantly lower levels of HLA‐DR compared to the LPS groups (*p*_LPS vs. Del_ = 0.0225, *p*_LPS vs. Ram_ = 0.0291, and *p*_LPS vs. MIX_ = 0.0240). CD80 expression was significantly higher in the LPS groups compared to Ram in the HCs' macrophages (*p* = 0.0344) and LPS versus Del in RA‐derived macrophages (*p* = 0.0256).

#### 4.7.3 The Expression Levels of CD14, CD80, and HLA‐DR Surface Markers in the M1 Macrophage Population of Healthy and RA Groups

Among the M1 macrophage population, all probiotic‐treated groups expressed lower levels of CD14, CD80, and HLA‐DR markers compared to the LPS‐treated groups. In RA patients, the probiotic‐treated groups, including Del (*p* = 0.0414) and Ram (*p* = 0.0205), demonstrated significantly reduced levels of CD14 compared to the untreated group. Additionally, all probiotic‐treated groups in RA patients showed decreased CD14 expression relative to LPS‐treated macrophages (*p*_Del_ = 0.0005, *p*_Ram_ = 0.0002, and *p*_Mix_ = 0.0055) (Figure 7B). Similarly, in healthy controls, probiotic‐treated groups exhibited significantly lower levels of CD14 than the LPS‐treated group (*p*_Del_ = 0.0010, *p*_Ram_ = 0.0004, and *p*_Mix_ = 0.0003). Comparison between untreated and LPS‐treated groups showed that CD80 expression was significantly lower in probiotic groups of HC (*p*_Del vs. LPS_ = 0.0014, *p*_Mix vs. LPS_ = 0.0006, and *p*_Ram vs. LPS_ = 0.0094) and RA patients (*p*_Del vs. LPS_ = 0.0324, *p*_Ram vs. LPS_ = 0.0011, and *p*_Mix vs. LPS_ = 0.0100). For HLA‐DR expression, significant differences were observed in the Unt vs. Ram (*p* = 0.0020) and Unt vs. Mix (*p* = 0.0443) groups in HCs, as well as between the untreated and Mix groups in RA patients (*p* = 0.0486). Comparisons between LPS‐treated and probiotic groups were significant only in the *L. delbrueckii* ‐treated group in HCs (*p* < 0.05) (Figure 7B).

#### 4.7.4 Evaluation of the Expression of CD14, CD80, and HLA‐DR Surface Markers in M2 Macrophage Populations of Healthy and RA Subjects

A comparison between LPS‐treated and untreated groups revealed that LPS treatment significantly increased the expression of CD14, CD80, and HLA‐DR markers in both control subjects (*p*_CD14_ = 0.0001, *p*_CD80_ < 0.0001, *p*_HLA‐DR_ = 0.0038) and RA patients (*p*_CD80_ = 0.0029) (Figure 7C). Among the M2 subpopulation of HC macrophages, certain probiotic‐treated groups expressed significantly lower levels of CD14 (*p*_Del vs. Unt_ = 0.0281, *p*_Ram vs. LPS_ < 0.0001, *p*_Mix vs. LPS_ < 0.0001), CD80 (*p*_Del vs. LPS_ = 0.0010, *p*_Ram vs. LPS_ < 0.0001, *p*_Mix vs. LPS_ = 0.0026), and HLA‐DR (*p*_Mix vs. Unt_ = 0.0290, *p*_Del vs. LPS_ = 0.0002, *p*_Ram vs. LPS_ < 0.0001, *p*_Mix vs. LPS_ < 0.0001) compared to both LPS‐treated and untreated groups. In RA patients, groups treated with Del (*p* = 0.0163) and Ram (*p* = 0.0387) expressed significantly lower levels of CD14 than untreated groups. Additionally, probiotic‐treated RA patients expressed reduced levels of CD14 (*p*_Del_ < 0.0001, *p*_Ram_ < 0.0001, *p*_Mix_ = 0.0307) and CD80 (*p*_Ram_ = 0.0087, *p*_Mix_ = 0.0008) compared to LPS‐treated groups (Figure 7C).

#### 4.7.5 Expression of CD14, CD80, and HLA‐DR in RA Patients and Healthy Controls

The comparison of CD14 expression in the M0 subpopulation between RA patients and HC showed that RA patients expressed higher levels of CD14 than HC. These differences were statistically significant in the Del (*p* < 0.0157) and Mix (*p* < 0.0106) treatment groups (Figure 7). However, in the M1 subpopulation, no significant differences were observed between RA patients and HC. In the M2 subpopulation, RA patients expressed significantly elevated levels of CD14 compared to controls across all treatment groups, with *p* values < 0.0001 for the LPS, Ram, and Del treatment groups, and *p* values of 0.0001 and 0.0002 for the Mix and untreated groups, respectively (Figure 7).

Comparisons of CD80 expression in the M0 subpopulation between RA patients and HC showed that although RA patients expressed higher levels of CD80 in the Del and Mix groups, these levels were significantly lower than those observed in the controls (*p* values for Del and Mix were 0.00175 and 0.0106, respectively) (Figure 7). In the M1 subpopulation, no significant differences were found between the treated groups of RA patients and HC. In the M2 subpopulation, all treated groups of RA patients expressed significantly higher levels of CD80 compared to HC (*p* values for Untreated, Del, Ram, Mix, and LPS < 0.0001) (Figure 7).

Comparisons of HLA‐DR expression in the M0 subpopulation between RA patients and HC showed that RA patients expressed higher levels of HLA‐DR than the controls. However, these differences were statistically significant in the Del, Ram, and Mix treatment groups, with *p* values of 0.0138, 0.038, and 0.0459, respectively (Figure 7). In the M1 subpopulation, the Ram (*p* = 0.0011) and LPS (*p* = 0.0090) treatment groups of RA patients expressed significantly higher HLA‐DR levels than their HC counterparts. In the M2 subpopulation of RA patients, all treatment groups expressed significantly elevated levels of HLA‐DR compared to HC, with *p* values for LPS and Del being < 0.0001, and for Ram and Mix being 0.0001 and 0.0002, respectively (Figure 7). Notably, in the M0 subpopulation, CD80, CD14, and HLA‐DR expression levels among RA patients were similar to those of healthy individuals. The comparable expression levels of CD80 and CD14 were also observed in the M1 subpopulations of both RA patients and healthy controls.

#### 4.7.6 Gene Expression Analysis of Cytokines

**FIGURE 8:**
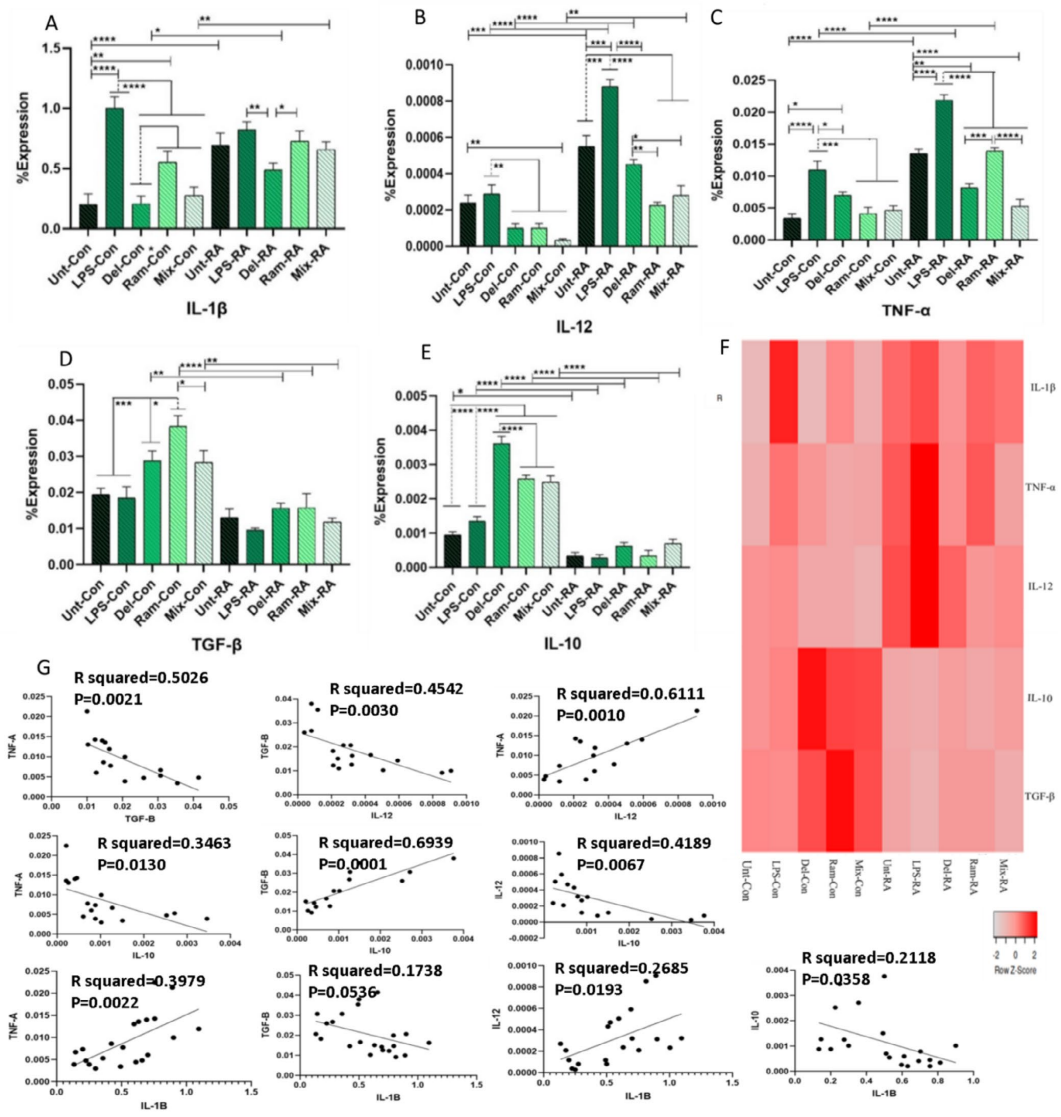
The real‐time PCR results of IL‐1β, TNF‐α, IL‐12, IL‐10, and TGF‐β genes in macrophages of healthy control (HC) and rheumatoid arthritis (RA) patients. (A) Except for comparing Ram‐con and unt‐con, probiotic treatment did not significantly alter IL‐1β levels compared to untreated groups. However, probiotics resulted in lower IL‐1β levels than LPS groups in RA and HCS macrophages. Further comparisons indicated that untreated, Del, and mixed RA patients exhibited significantly higher IL‐1β levels than controls. (B) Probiotic treatment reduced the expression of IL‐12 compared to untreated and LPS groups. In counterpart comparisons, RA macrophages expressed more IL‐1β than control macrophages. (C) Except for Del and mixed groups in RA macrophages, all probiotic groups exhibited insignificantly higher IL‐12 levels than untreated groups. Still, none reached levels equal to or higher than those in LPS groups. Counterpart comparisons revealed that RA macrophages expressed higher IL‐12 levels than controls, with significant differences observed in Ram, LPS, and untreated groups. (D) Probiotic groups demonstrated higher TGF‐β levels than untreated and LPS groups, with significance noted only in control groups. Counterpart comparisons showed that HCS macrophages expressed higher TGF‐β levels than controls, which were significant in Ram, LPS, and untreated groups. (E) Probiotic‐treated groups exhibited higher IL‐10 levels than untreated and LPS groups, with significance observed only in control groups. (F) A heat map visualizing the expression levels of IL‐1β, TNF‐α, IL‐12, IL‐10, and TGF‐β genes across different treatment groups of RA patients and healthy donors. (G) Correlations between the expressions of various cytokines Untreated (Unt), *Lactobacillus delbrueckii* (Del), and *Lactobacillus rhamnosus* (Ram). The bars are presented with mean ± standard deviation (SD) values. Different letters in the figure indicate significant differences between groups (*p* < 0.05). The statistical analysis was conducted using a one‐way analysis of variance (ANOVA) test: *p < 0.05, **p ≤ 0.01, ***p ≤ 0.001, and ****p < 0.0001.

To evaluate the effects of probiotics on macrophage polarization, the RNA expression levels of IL‐12, IL1‐β, TNF‐α, IL‐10, and TGF‐β were analyzed by quantitative real‐time PCR. In this study, the expression of IL1‐β was higher in the LPS‐treated groups of RA patients and HC (*p* < 0.0001) than in the untreated groups. Probiotic‐treated groups expressed lower levels of IL‐1 β than LPS‐treated groups in RA patients (*p*_Del_ = 0.0022) and HC (*p*_Del, Ram, and Mix_ < 0.0001). Comparing the untreated and probiotic‐treated groups, probiotics decreased IL‐1β levels in RA patients. However, in HC, probiotics increased the expression of IL‐1β compared to the untreated group, which was significant only in the Ram‐treated group (*p* = 0.0014) (Figure 8A).

LPS‐treated groups increased the expression of IL‐12 in RA patients (*p* = 0.0001) and HC. Probiotic treatment decreased the expression of IL‐12 compared to untreated groups (in RA patients, *p*_Ram_ = 0.0001, *p*_Mix_ = 0.0008 and in HC, *p*_Mix_ = 0.0069) and LPS‐treated groups (in RA patients, *p*_Del, Ram, Mix_ < 0.0001 and HC, *p*_Del_ = 0.0063, *p*_Ram_ = 0.0064, *p*_Mix_ = 0.0005) (Figure 8B).

Evaluation of TNF‐α expression showed that LPS treatment of macrophages significantly increased the expression of this cytokine in both RA patients and HC (*p* < 0.0001). In HC, probiotic treatment of macrophages decreased TNF‐α expression levels more than in untreated (*p*_Del_ = 0.0251) and LPS‐treated groups (*p*_Del_ = 0.0132, *p*_Ram_ = 0.0001, *p*_Mix_ = 0.0001). Notably, in RA patients, probiotics decreased TNF‐α expression levels compared to untreated (*p*_Del_ = 0.0012 and *p*_Mix_ = < 0.0001) and LPS‐treated groups (*p*_Del, Ram, and Mix_ < 0.0001) (Figure 8C).

LPS treatment decreased the secretion of IL‐10 and TGF‐β in both RA patients and HC, but it was not statistically significant. Probiotic treatment reduced the expression of IL‐10 and TGF‐β in RA patients and HC, but this reduction was statistically significant only in controls (for IL‐10, *p*_Del, Ram, Mix vs. Unt_ < 0.0001 and *p*_Del, Ram, Mix vs. LPS_ < 0.0001 and for TGF‐β, *p*_Ram vs. Unt_ = 0.0001 and *p*_Ram vs. LPS_ < 0.0001) (Figure 8D,E).

A positive correlation was observed between IL‐1β and the other inflammatory cytokines IL‐12 (*p* = 0.0193) and TNF‐α (*p* = 0.0022). Conversely, there was a negative correlation between the expression of IL‐1β and anti‐inflammatory cytokines, including IL‐10 (*p* = 0.0358) and TGF‐β (*p* = 0.0536). In addition, a negative correlation was found between the expression of IL‐10 and IL‐12 (*p* = 0.0067) and TNF‐α (*p* = 0.0130). In contrast, a positive correlation was observed between the expression of IL‐10 and TGF‐β (*p* = 0.0001) (Figure 8G).

#### 4.7.7 Comparison Between the Expression of IL‐10, TGF‐β, IL‐1, IL‐12, and TNF‐α in RA Patients and HC

Comparison between RA patients and HC showed that HC macrophages expressed higher levels of TGF‐β than RA patients, which was significant in Del (*p* = 0.0085), Ram (*p* = 0.0001), and Mix (*p* = 0.0011) groups. This comparison for IL‐10 expression showed that HC expressed significantly higher levels of this cytokine than RA patients in all groups (*p*_Untreated, LPS, Del, Ram, Mix_ < 0.0001) (Figure 8).

Comparisons between all treated groups of RA patients and HC showed that RA patients expressed significantly higher levels of IL‐12 than HC in LPS (*p* < 0.0001), Del (*p* < 0.0001), and untreated (*p* = 0.0002) groups. More comparisons between RA patients and HC showed that, except for LPS groups showing similar expression, RA patients expressed higher levels of IL‐1β than HC, which were significant in Del (< 0.0001), Untreated (0.0123), and Mix (0.0018) groups. For TNF‐α expression, RA patients expressed higher levels of this cytokine than controls in all treated groups, which were statistically significant in LPS, Ram, and Untreated groups (*p*_LPS, Ram, Unt_ < 0.0001) (Figure 8).

## 5 Discussion

### 5.1 Probiotics Suppressed the Expression of CD14 on Macrophages

CD14, or monocyte differentiation antigen, is a marker expressed on monocytes and macrophages. It plays a crucial role in phagocytosis, binding to LPS, and stimulating the secretion of IL‐6, IL‐1, and TNF‐α. Additionally, CD14 functions as a co‐receptor for TLR2 and TLR4 (Wu et al. 2019; Schulz et al. 2019). This marker is distinctly expressed in different classes of monocytes, with higher expression in intermediate and classical monocytes. These monocytes are more likely to differentiate into M1 macrophages, while nonclassical monocytes exhibit lower expression and are more susceptible to differentiating into M2 macrophages (Rana et al. 2018). The quantity of classical and intermediate monocytes is greater than that of nonclassical monocytes in patients with rheumatoid arthritis. In this study, to evaluate the purity of monocytes and the efficacy of the monocyte isolation process performed by the plastic adherence method, we assessed the expression of CD14 and CD45 among the isolated cells (Rana et al. 2018).

In this study, M1 had the highest levels of CD14 expression among the macrophage subpopulations. Other research has demonstrated that cytokines or substances that promote the polarization of M0 macrophages to M1 can enhance CD14 expression. (Rana et al. 2018) showed that treating alveolar macrophages with LPS, a substance that promotes the differentiation of monocytes into M1 macrophages, can also enhance CD14 expression on these M1 macrophages. CD14 plays a crucial role in initiating phagocytosis. Meanwhile, LPS treatment of macrophages can elevate the expression of IL‐6, IL‐1β, and INF‐γ through the stimulation of TLR‐2 and TLR‐4 (Sladek and Rysanek 2009). Studies have shown that anti‐inflammatory agents such as IL‐4, promoting the differentiation of macrophages into the M2 phenotype, can reduce CD14 expression (Isidro and Appleyard 2016). Consequently, the reduction in CD14 expression led to a decrease in the secretion of TNF‐α, IL‐1β, INF‐γ, and IL‐6 through TLR‐dependent pathways (Yang et al. 2016). In our study, the M0 subpopulation had higher levels of CD14 expression compared to M2 macrophages. However, as monocytes differentiated into macrophages, CD14 expression gradually decreased but remained high. Similarly, Research has demonstrated that the differentiation of monocytes into macrophages, stimulated by GM‐CSF treatment, is associated with a reduction in CD14 expression in macrophages (Kruger et al. 1996).

In this study, the LPS‐treated groups had higher levels of CD14 expression compared to the untreated groups. According to research conducted by Yang et al., there are two CpG islands within the CD14 gene: one located in the promoter region and the other within the coding sequence of CD14. LPS treatment of macrophages can activate the enzyme DNA methyltransferase 3 (DNMT3a), leading to the methylation of only the CpG island in the coding sequence (Yang et al. 2016). Also, in our study, all probiotic treatment groups expressed lower levels of CD14 than both untreated and LPS‐treated groups. The probiotics *L. delbrueckii* and *L. rhamnosus* demonstrated anti‐inflammatory properties. Consistent with our findings, the study conducted by Boyle et al. (2011) revealed that the supplementation of 125 pregnant women with *L. rhamnosus* GG for 36 weeks significantly reduced the levels of secretory CD14 in breast milk. In another study, the addition of cell‐free fermented milk supernatant of *L. paracasei* resulted in a significant decrease in CD14 expression in monocytes (Tulini et al. 2015).

### 5.2 Probiotics Dwindled the Expression of CD80 on Macrophages

CD80, also known as B7.1, is a marker found on antigen‐presenting cells, including macrophages and monocytes (Ke et al. 2002). This marker serves as a ligand for CD28 or CTLA‐4 on T cells. When the TCR binds to MHC, it initiates T cell activation, the first stimulatory signal for T cells. The CD28‐CD80 complex then exerts co‐stimulatory activity, recognized as the second stimulatory signal for T cells (Bashian et al. 1997). The coexistence of primary and secondary stimulatory signals in T cells results in the secretion of IL‐2 by these cells. The binding of CD80 to CTLA‐4 has a significantly higher affinity, which causes the dissociation of CD80 on macrophages, thereby functioning as a regulator of the immune response (Wongjitrat et al. 2013). In this study, the M1 subpopulation of macrophages had significantly higher levels of CD80 expression compared to the M0 and M2 subpopulations. Several studies have demonstrated that various inflammatory conditions, such as IL‐6, LPS, and INF‐γ, promote macrophage polarization toward the M1 phenotype, leading to a substantial increase in CD80 expression on macrophages (Jiménez‐Uribe et al. 2019). M1 macrophages, the most effective cells in T cell priming, predominantly express CD80. In contrast, M2 macrophages had the lowest levels of CD80 expression. Consistent with our findings, other studies have demonstrated that anti‐inflammatory substances, including IL‐4, IL‐13, IL‐10, and glucocorticoids, can stimulate M2 macrophages while simultaneously suppressing CD80 expression in these cells (Shiratori et al. 2018; Lasswell et al. 2017). In this study, M0 macrophages also expressed lower levels of CD80 than M1 but higher levels than M2 macrophages. In the research conducted by Jaguin et al. (2013), M0 macrophages expressed levels of CD80 that were approximately similar to those of IL‐4‐treated macrophages.

There is a connection between the stimulation of the CD14 marker on macrophages and the expression of the CD80 marker on these cells. By stimulating CD14 and TLRs, the transcription factor NF‐κB is activated, which enhances the transcription of CD80 in macrophages (Berrebi et al. 2003). Both LPS, found in the cell wall of Gram‐negative bacteria, and lipoteichoic acid (LTA), present in the cell wall of Gram‐positive bacteria such as *Lactobacillus* spp., can stimulate CD14; however, LPS is more effective (Berrebi et al. 2003; Watanabe et al. 2023). This may be the primary reason why LPS‐treated groups expressed significantly higher levels of CD80 compared to untreated and probiotic‐treated groups. Meanwhile, LTA, a weak stimulator of CD14, is found in *L. delbrueckii* and *L. rhamnosus* . Although it can weakly stimulate CD14, probiotic‐treated groups showed only a nonsignificant increase in CD80 levels compared to untreated groups (Poxton 2014).

### 5.3 Probiotics Decrease the Expression of HLA‐DR on Macrophages

HLA‐DR is an MHC class II molecule expressed on antigen‐presenting cells that can stimulate CD4+ T cells (Holzbecher et al. 1984). In this study, M1 macrophages expressed higher HLA‐DR levels than M0 and M2 macrophages. Consistent with our findings, (Jaguin et al. 2013) demonstrated that M1 macrophages expressed elevated levels of HLA‐DR relative to M2 macrophages. In this study, LPS treatment increased the expression of HLA‐DR on macrophages. Other studies have shown that treatment of THP‐1 macrophages with IFN‐γ and LPS results in M1 macrophage differentiation and increased HLA‐DR expression (Portillo et al. 1989; Popěna et al. 2018). Inflammatory conditions, such as those induced by IFN‐γ, can enhance the expression of class II transactivator (CIITA), the primary transcription factor for HLA‐DR, and activate the tyrosine kinases JAK1 (Janus kinase 1) and JAK2. This activation leads to the phosphorylation and dimerization of the signal transducer and activator of transcription 1 (STAT1). The translocation of STAT1 to the nucleus promotes the expression of CIITA (Portillo et al. 1989).

In this study, the M2 macrophage subpopulation had the lowest levels of the HLA‐DR marker. Numerous studies have shown that anti‐inflammatory agents such as TGF‐β, IL‐13, IL‐10, and IL‐4 are capable of stimulating macrophage polarization to the M2 phenotype and suppressing HLA‐DR expression in macrophages (Popěna et al. 2018; Won et al. 2005; Yao et al. 2004). These studies highlight the association between macrophage M2 polarization and the decrease of HLA‐DR.

In this study, the M0 subpopulation expressed lower levels of HLA‐DR than M1 macrophages. Treatment of THP‐1 macrophages with IFN‐γ and LPS led to the polarization of M1 macrophages and an increase in HLA‐DR expression. In contrast, treatment with IL‐4 and IL‐13 resulted in a negligible increase in HLA‐DR expression in M2 macrophages (Popěna et al. 2018). Our findings indicate that the M0 subpopulation had significantly higher levels of HLA‐DR expression than M2 macrophages, suggesting a more M1‐like phenotype. Probiotic‐treated groups in our study dominantly exhibited lower levels of HLA‐DR compared to both LPS‐treated and untreated groups, highlighting probiotics' anti‐inflammatory and immunoregulatory properties. It significantly influences the antigen‐presenting function of macrophages, a crucial process in initiating autoantibody production in autoimmune diseases such as RA. The impact of probiotics on the stimulation of T helper cells is worth studying in the future.

### 5.4 Probiotics Boosted the Expression of Anti‐Inflammatory Cytokines

In our study, IL‐10 expression levels were higher in the probiotic‐treated groups compared to the untreated and LPS‐treated groups. Similarly, Fernández‐Llanio Comella et al. (2016) indicated that RA patients with *L. casei* supplementation were associated with increased IL‐10 expression. Treatment of THP‐1 macrophages with extracellular vesicles derived from *L. plantarum* also elevated IL‐10 expression (Kim et al. 2020). Yoshikawa et al. demonstrated that *L. rhamnosus* and *L. paracasei* can induce IL‐10 expression in macrophages. These bacteria can bind to carbohydrate receptors on macrophages, including Dectin‐1, Dectin‐2, and Mincle, stimulating macrophage phagocytosis, which leads to NOD2‐dependent immune responses and increased IL‐10 expression (Yoshikawa et al. 2021). In our study, macrophages isolated from RA patients expressed significantly lower levels of IL‐10 than those from HC across all treatment groups, indicating the importance of this cytokine in RA autoimmunity. Shrivastava et al. (2015) found that RA patients had significantly lower levels of IL‐10 in their serum samples than HC. A study on LPS‐stimulated THP‐1 macrophages revealed that treatment with heat‐killed *L. rhamnosus* and *L. plantarum* increased IL‐10 expression (Magryś and Pawlik 2023).

This study also showed that treating macrophages with LPS resulted in a sharp decrease in the expression of TGF‐β in these cells. In parallel, the study by Zhang et al. (2017) showed that LPS treatment led to reduced TGF‐β expression in macrophages. Additionally, another study found that treatment with anti‐inflammatory cytokines, including IL‐13 and IL‐4, polarized macrophages into M2a macrophages, which are characterized by the overexpression of IL‐10 and TGF‐β (Shapouri‐Moghaddam et al. 2018). Similarly, reported that TGF‐β treatment decreased the expression of CD14 and HLA‐DR in macrophages (Carta et al. 2021). Collectively, these studies suggest that M1 macrophages secrete lower levels of TGF‐β. Furthermore, probiotic treatment of macrophages increased TGF‐β expression in RA patients and healthy controls, although this increase was statistically significant only in the control group. Acosta et al. showed that the S‐layer of *L. brevis* , by binding to minicles on macrophage cells, could activate caspase recruitment domain‐containing protein‐9 (CARD9) and spleen‐associated tyrosine kinase 1 (Syk). This activation led to a significant suppression of TNF‐α and IL‐6, along with an increase in IL‐10 and TGF‐β (Prado Acosta et al. 2021).

### 5.5 Probiotics Attenuated the Expression of Inflammatory Cytokines

LPS treatment of macrophages was associated with an increase in IL‐1β expression. Studies have shown that LPS can bind to CD14 and stimulate TLR4, leading to the activation of NF‐κB and an increase in IL‐1β expression (Bent et al. 2018; Metchnikoff and Prize 2015; Wang et al. 2020). In contrast, probiotic treatment of macrophages suppressed IL‐1β expression (Jang et al. 2024). Similarly, Sichetti et al. (2018) showed that treating THP‐1 macrophages with *L. rhamnosus* , *B. lactis*, and *B. longum* resulted in decreased IL‐1β. The other study found that *L. rhamnosus* could decrease the expression of TLR4 and MYD88, thereby inactivating the transcription of IL‐1β in LPS‐pretreated cells. Additionally, *L. rhamnosus* could inactivate the expression of mitogen‐activated protein kinase (MAPK) and NF‐κB subunits (Liu et al. 2022). Ultrasonicated *L. rhamnosus* suspension inhibited the activation of the NF‐κB/c‐Fos/NFATc1 pathway in α‐MEM, M‐CSF, and RANKL‐pretreated bone marrow‐derived macrophages or RAW264.7 macrophages (Fu et al. 2024). Thus, *L. rhamnosus* can suppress IL‐1β expression.

This study also showed that LPS‐treated macrophages expressed higher levels of IL‐12 compared to M1 macrophages. LPS treatment not only stimulates IL‐12 expression but also enhances the polarization of M1 macrophages. LPS can induce rearrangement of nucleosome 1, allowing the LAP/(C/EBPβ) transcription factor to bind to its site on the promoter of the IL‐12b gene. Additionally, LPS activates the AP‐1 transcription factor, which further enhances the expression of the IL‐12β gene. Furthermore, LPS binds to CD14 and TLR‐4, activating MyD88, which subsequently activates the AP‐1, NF‐κB, and C/EBPβ transcription factors, leading to increased IL‐12a and IL‐12B protein expression (Zheng et al. 2016). Treatment of macrophages with IL‐4 and prostaglandin E2, two substances that promote M2 polarization, can activate the repressor of the IL‐12β gene known as GAP‐1 (Shapouri‐Moghaddam et al. 2018; Zheng et al. 2016). In this study, treatment of macrophages with probiotics decreased IL‐12 expression in both LPS‐treated and untreated groups. Consistent with our findings, Matsubara et al. (2017) reported that treating THP‐1 macrophages with *L. rhamnosus* significantly reduced IL‐12 expression. Notably, significant differences in IL‐12 expression levels between healthy controls HC and RA patients indicate the importance of this cytokine in RA (Zheng et al. 2016).

TNF‐α is an essential inflammatory cytokine. The study conducted by Dichtl et al. (2022) showed that TNF‐α can inhibit the expression of various genes, including those involved in tissue repair, by binding to its type 1 receptor and activating the JNK pathway in tissue‐repairing M2 macrophages. Consequently, this cytokine can inhibit M2 macrophages and hinder tissue repair in rheumatoid arthritis, highlighting its importance in the disease's progression. The severity of rheumatoid arthritis is directly correlated with serum and synovial TNF‐α levels, and numerous drugs targeting this cytokine have been developed for the treatment of the disease (Moelants et al. 2013). This study demonstrated that treating macrophages with LPS increased TNF‐α expression. Similarly, Huang et al. (2019) found that treating THP‐1 macrophages for 48 h significantly elevated TNF‐α expression and promoted polarization toward the M1 phenotype. The TNF‐α promoter contains specific binding sites for LITAF (LPS‐induced TNF‐α factor), NF‐κB, and AP‐1. LPS treatment results in the dimerization and activation of LITAF and STAT6 (B). Additionally, LPS binds to CD14 and activates TLR‐4, which in turn leads to the activation of NF‐κB (Shio et al. 2014; Tang et al. 2005). Our study also showed that in all five treatment groups, patients with rheumatoid arthritis exhibited higher levels of TNF‐α compared to healthy individuals, underscoring the significance of this cytokine in the pathogenesis of rheumatoid arthritis. Treatment with *L. delbrueckii* probiotics, as well as a combination of two probiotics, significantly reduced the expression of TNF‐α in comparison to the untreated group. Consistent with our findings, the research conducted by Jang and colleagues also indicated that treating macrophages with *L. brevis* could decrease TNF‐α expression by inhibiting the transcription factor NF‐κB and promote differentiation toward the M2 phenotype in macrophages (Jang et al. 2013). In this study, the groups treated with probiotics expressed lower TNF‐α levels than those treated with LPS, with *Lactobacillus* demonstrating a significant reduction. Another study also showed that *L. rhamnosus* could decrease TNF‐α expression in previously stimulated feline macrophage cell lines (Fcwf‐4) (Jang et al. 2024).

Cytokines between LPS‐treated and probiotic‐treated groups and between untreated groups and probiotic‐treated groups were significant in many cases; the overall expression of these cytokines was marginal. This low expression level may not significantly impact the macrophage microenvironment. The reduced cytokine levels might be attributed to the fact that in this in vitro study, monocytes were directly isolated from PBMCs and, without the addition of any cytokines or other stimuli, spontaneously differentiated into macrophages within 5 days before being treated with LPS or probiotics. In contrast to the in vivo microenvironment, where macrophages are exposed to exosomes, cytokines, and other microbiota that epigenetically alter macrophage cytokine secretion, this in vitro study isolated macrophages from external factors, allowing for a focused examination of the effects of LPS and probiotics. Therefore, it appears that in vivo studies investigating the immunomodulatory effects of probiotics on macrophages are necessary.

## 6 Study Limitations and Future Studies

The present in vitro study has limitations, including a small sample size. This limitation has been observed in numerous studies investigating the effects of various *Lactobacillus* species on macrophages (Kim et al. 2020; Yoshikawa et al. 2021; Esmaeili et al. 2018; Rodes et al. 2013; Shida et al. 2009; Matsubara et al. 2017; Lin et al. 2008). Therefore, conducting more in vitro studies with larger sample sizes or meta‐analysis studies is highly recommended. In addition, macrophages are cells in various tissues, making them susceptible to cytokines, exosomes, and microbiota within their microenvironment. These external influences can affect macrophage gene expression and alter their behavior in response to probiotics. Therefore, in vivo studies that monitor the effects of probiotics on macrophages in various microenvironments could be essential. It is also recommended that the expression levels of cytokines be evaluated not only by the qRT‐PCR method but also by the enzyme‐linked immunosorbent assay (ELISA) method, measuring the protein products. Moreover, it would be intriguing to investigate the effects of *Lactobacillus* pre‐treatment on the outcomes of LPS posttreatment in human monocyte‐derived macrophages from healthy individuals and RA patients (Kim et al. 2021, 2022; Yamazaki et al. 2020; Di Chiano et al. 2024; Qi et al. 2020). Aside from the markers examined in this study, including CD14, CD80, HLA‐DR, IL‐10, TGF‐β, TNF‐α, IL‐12, and IL‐1β, M1 and M2 macrophages distinctly express other markers, including inducible nitric oxide synthase (iNOS) and Arginase 1, and examining them in the studies is suggested.

## 7 Conclusion

The data suggest that *L. delbrueckii* and *L. rhamnosus* are anti‐inflammatory and immunomodulatory probiotics that do not exhibit cytotoxicity toward macrophages. These probiotic bacteria regulate the immune system by influencing macrophage polarization. They can initiate autoimmune processes by presenting autoantigens to T cells and producing pro‐inflammatory cytokines. This study demonstrated that these probiotics can promote the polarization of macrophages toward the M2 subpopulation, which is characterized by high expression levels of IL‐10 and TGF‐β and low expression levels of CD14, CD80, and HLA‐DR, IL‐1β, IL‐12, and IFN‐γ. Therefore, *L. delbrueckii* and *L. rhamnosus* may benefit both prophylactic and therapeutic purposes. They may decrease CD14 receptor‐mediated endocytosis, reduce the effective presentation of autoantigens to T cells by downregulating CD80 and HLA‐DR, and diminish the production of inflammatory cytokines, thereby preventing or alleviating autoimmune diseases such as RA.

## Author Contributions

**Parisa Ahmadi:** methodology (equal), project administration (equal), software (equal), writing – original draft (equal). **Mahmoud Mahmoudi:** project administration (equal), supervision (equal). **Houshang Rafatpanah:** funding acquisition (equal). **Zahra Rezaieyazdi:** project administration (equal). **Maryam Ahmadi‐Khorram:** writing – review and editing (equal). **Zahra Javanmardi:** project administration (equal). **Nafiseh Sadat Tabasi:** project administration (equal). **Seyed‐Alireza Esmaeili:** conceptualization (equal), methodology (equal), project administration (equal), supervision (equal), writing – review and editing (equal).

## Conflicts of Interest

The authors declare no conflicts of interest.

## Supporting information


Figures S1–S2

## Data Availability

The data will be made available on request.
