# Supplementary material for: The Impacts of Lactobacillus delbrueckii and Lactobacillus rhamnosus to Promote In Vitro Anti‐Inflammatory Profile of RA‐Macrophages
Source: Food Sci Nutr. 2025 Mar 16;13(3):e70068. doi: 10.1002/fsn3.70068 (PMC11911130; doi:10.1002/fsn3.70068)
Supplement: Supplementary file 1 — Figures S1–S2 [file FSN3-13-e70068-s001.docx]

## Supplementary File


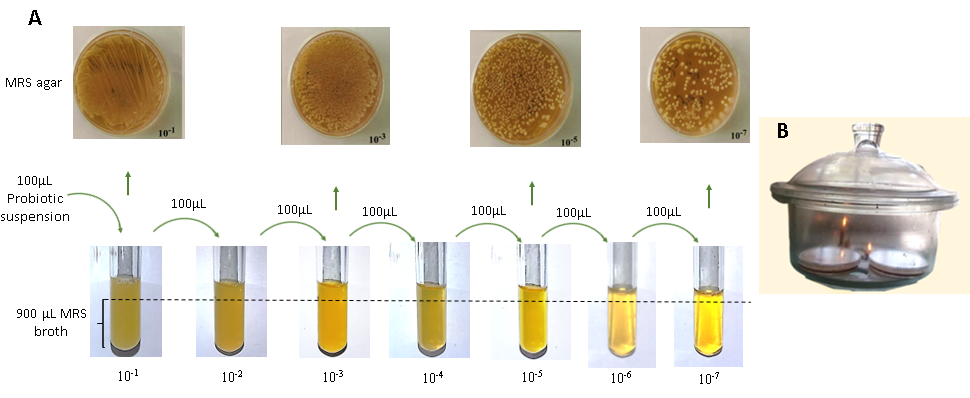


**Figure S1 Culturing of *Lactobacillus* *Delbruecki* and *Lactobacillus rhamnosus* in MRS broth and dilution of them through serial dilution method in 900μL MRS broth then re-culturing of each dilution on MRS agar (A) in an anaerobic jar (B).**


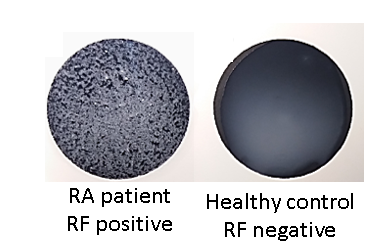


**Figure S2 the result of the rheumatoid factor rapid test**
